# Supplementary figures and images for: Identification of Potential miRNA–mRNA Regulatory Network Associated with Growth and Development of Hair Follicles in Forest Musk Deer
Source: Animals (Basel). 2023 Dec 15;13(24):3869. doi: 10.3390/ani13243869 (PMC10740511; doi:10.3390/ani13243869)

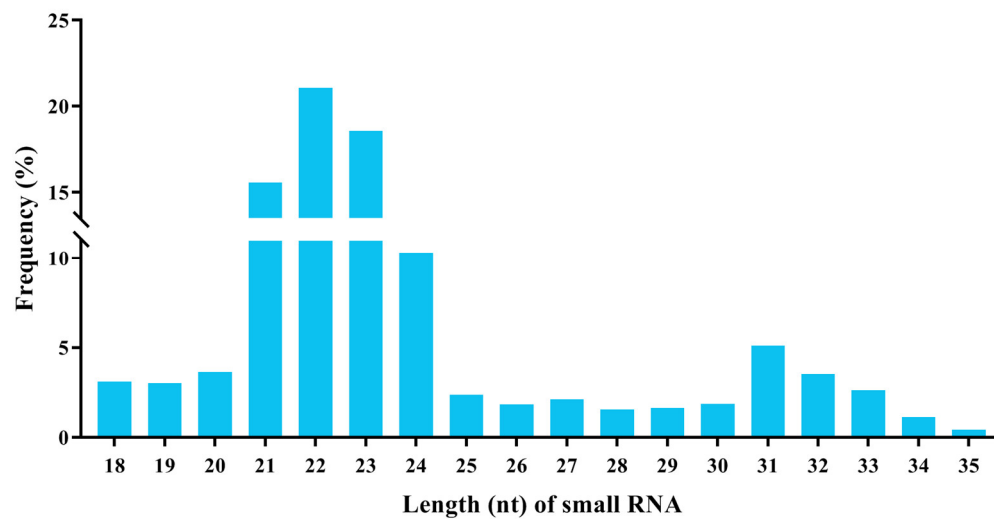

**Figure S1.** The length distributions of sRNA of hair follicles at the anagen and catagen stage.

Supplement: Supplementary file 1 [file animals-13-03869-s001.zip › Supplementary Figure S1.pdf]
